# Sirtuin 1 in Chronic Kidney Disease and Therapeutic Potential of Targeting Sirtuin 1

**DOI:** 10.3389/fendo.2022.917773

**Published:** 2022-06-20

**Authors:** Jiayi Yan, Jue Wang, John Cijiang He, Yifei Zhong

**Affiliations:** ^1^ Division of Nephrology, Longhua Hospital, Shanghai University of Traditional Chinese Medicine, Shanghai, China; ^2^ Department of Medicine, Division of Nephrology, Icahn School of Medicine at Mount Sinai, New York, NY, United States

**Keywords:** kidney, SIRT1, vascular calcification, chronic kidney disease, HIV infection

## Abstract

The incidence and prevalence of chronic kidney disease (CKD) continue to increase worldwide remaining as a major public health burden. CKD eventually progresses to end-stage kidney failure and patients with CKD have high morbidity and mortality. Sirtuin 1 (SIRT1), a NAD+-dependent deacetylases, has significant renal protective effects through its regulation of fibrosis, apoptosis, and senescence, oxidative stress, inflammation and aging process. The renal protective effects of Sirt1 have been described in many kidney diseases such as diabetic kidney disease and HIV-related kidney disease. SIRT1 also has protective effects against vascular calcification and therefore could be developed as a therapy for both CKD and CKD complications. In this narrative review, we will give an overview of the recent progress on the role of SIRT1 and its downstream pathways in CKD. We will also discuss potential therapeutic approach by activating SIRT1-related pathway in patients with CKD. The purpose is to hope to provide some insights on the future direction of the research in the field of SIRT1 for CKD.

## Introduction

About 10% of the human population have chronic kidney disease (CKD), which have high incidence of cardiovascular disease and mortality (1). CKD is characterized by a progressive loss of kidney function with glomerular, tubular and vascular injuries and cardiovascular disease is the most important complication of CKD which is characterized by vascular calcification (2–4). Therefore, new therapies are urgently needed to halt the progression of CKD and prevent complications of CKD. A number of studies have shown that SIRT1, a nicotinamide adenine dinucleotide-dependent histone deacetylase, has pivotal roles on renal protection through reduction of oxidative stress, inflammation, and fibrosis (5–8). In addition, Sirt1 also inhibits apoptosis and regulates metabolism (9). Therefore, the activation of SIRT1 may imply a therapeutic strategy to improve the clinical outcome of CKD. This review focuses on the protective effects of SIRT1 against CKD progression and complications through its regulation of fibrosis, apoptosis, oxidative stress, inflammation and aging process.

## Expression of SIRT1 in the Kidney

SIRT1 is a highly conservative protein and contains 500 amino acid residues, which was first discovered in 1999 (10). SIRT1 is expressed in the kidneys and can protect and maintain normal kidney cell function by mediating in various physiological processes (10). SIRT1 is mainly resided in the nucleus where it facilitates nuclear cytoplasmic shuttling by regulating both nucleosome histone acetylation and the activity of several transcriptional factors (11–13). SIRT1 can also be found in the cytoplasm. A few studies showed SIRT1 inhibits TNF*α*-dependent transactivation of NF-*κ*B in TNF-α induced cytokine production in fibroblast cells and the expression of several proinflammatory genes are inhibited (14). SIRT1 is a negative regulator of p53. SIRT1 can deacetylate p53 and result in reduced cellular senescence and apoptosis after DNA damage and oxidative stress (15, 16).

## SIRT1 and Renal Fibrosis

Renal fibrosis is a major feature of progressive CKDs (3). Studies have focused on the role of SIRT1 for renal fibrosis. Deficiency of *Sirt1* in endothelial cells increases peritubular capillary rarefaction (17) and aggravates nephrosclerosis, *via* downregulation of matrix metalloproteinase-14, which indicates a role of SIRT1 in renal fibrosis (18). SIRT1 also regulates kidney fibrosis by inducing deacetylation of Smad4 and inhibiting TGF-β-mediated matrix metalloproteinase-7 expression in kidney tubular epithelial cells (19). Activation of SIRT1 can lead to attenuated renal fibrotic processes. SIRT1 reverses Smad3 acetylation and thereby inhibiting the profibrotic response of TGF-β1 *in vitro* and *in vivo* such as in unilateral-ureteric obstruction mouse model of renal fibrosis (20, 21). In addition, a Chinese herbal formula, Shen Shuai IIRecipe (SSR), significantly attenuates renal injury and fibrosis in the remnant kidneys (22). SSR could contribute to renal protection by up-regulating SIRT1/Smad3 deacetylation pathway and attenuating renal fibrosis in 5/6 nephrectomy model of CKD (22). Furthermore, tubular cell-specific overexpression of SIRT1 attenuates the progression of AKI to CKD transition through Smad4 deacetylation (19). Therefore, activation of SIRT1 could be a potential approach to develop anti-fibrosis therapy for CKD.

## SIRT1 in Different Kidney Diseases

SIRT1 plays a major role in diabetic kidney disease (DKD). In our previous studies, we also find SIRT1 can regulate NF-κB (p65) and STAT3 acetylation in DKD. Since SIRT1 expression is reduced in the diabetic kidney, the acetylation level of p65 and STAT3 is increased in diabetic kidneys (23). The loss of SIRT1 in the podocyte of diabetic db/db mice increases acetylation of p65 and STAT3 as a result of exacerbated proteinuria and kidney injury, likely through increased inflammatory response. Conversely, a bromodomain inhibitor, MS417, significantly attenuates proteinuria and kidney injury. MS417 is able to block acetylation-mediated association of p65 and STAT3 with bromodomain and extra-terminal domain (BET) proteins in the kidney cells from diabetic mice. These studies therefore indicate the critical role of SIRT1 against proteinuria and kidney injury by decreasing acetylation of p65 NF-κB and STAT3 in DKD (23). For further exploring the function of SIRT1, we generated inducible and reversible Sirt1-knockdown mice in a global, or podocyte-specific, or tubular-specific pattern. We find that knockout of SIRT1 either globally or specifically in podocytes induced more albuminuria and glomerulosclerosis in Adriamycin-induced nephropathy mouse model as compared to wild-type mice with Adriamycin-induced nephropathy. Knockdown of SIRT1 also induces more mitochondrial injury and cell senescence in the kidney cells in diabetic mice as compared with control diabetic mice (24). These results indicate a critical role of SIRT1 in the pathogenesis of DKD by regulating mitochondrial stress/injury and cell senescence of kidney cells.

**Figure 1:**
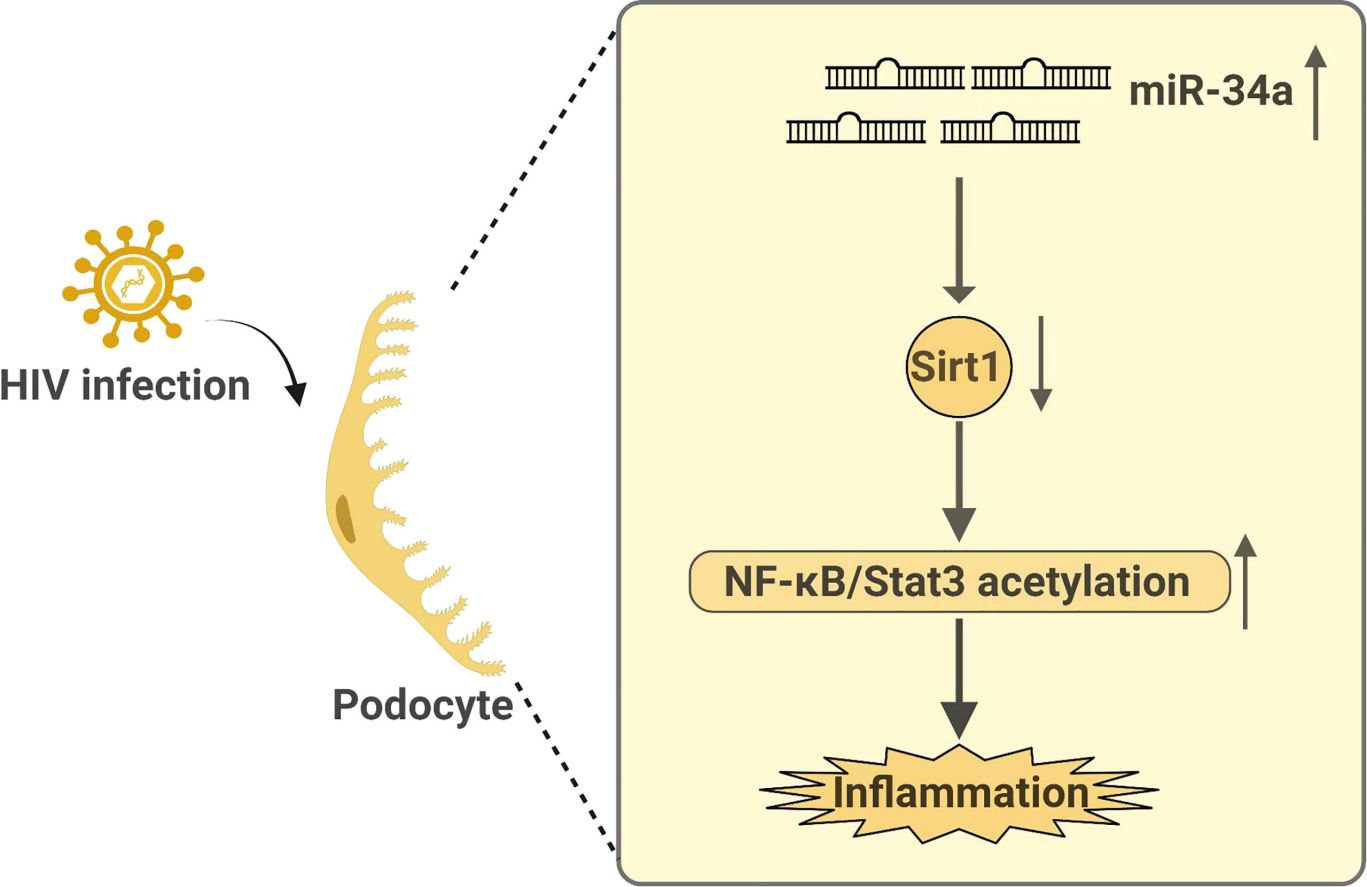
Summary on how HIV infection regulates Sirt1 expression and how Sirt1 mediates HIV infection-induced kidney disease.

Recently, we also demonstrate a role of SIRT1 in HIV-related kidney disease. We find that HIV infection suppresses SIRT1 expression in kidney cells, leading to increased acetylation and activation of NF-κB and STAT3, similar to those observed in DKD (25). Mechanistically, this is through HIV-induced miR-34a expression, which downregulates Sirt1 mRNA level. Sirt1 not only inhibits proinflammatory response but also HIV viral gene expression in podocytes and thereby attenuating HIV-induced podocyte injury as summarized in the **Figure 1** (25). Therefore, SIRT1 is also a key protector against HIV-mediated CKD pathogenesis. In addition, HIV infection and diabetes have a synergistic or additive effect on the progression of CKD (25). We believe that SIRT1, which is suppressed by both diabetes and HIV infection, plays a key role in mediating the synergistic effects between diabetes and HIV and may explain why HIV infection aggravates DKD progression.

The studies of Sirt1 in human CKD are limited. In our previous study, we find that both mRNA and protein levels of Sirt1 are reduced in glomeruli of patients with diabetic kidney disease (DKD) as assessed by qPCR and immunostaining (26). Consistent with this, we also show that acetylation of NF-kB and STAT3 is increased in human diabetic kidney (23). SIRT1 expression is also suppressed in the kidneys from HIV-infected patients (25). How Sirt1 expression and function are regulated in human CKD remains unclear. It has been shown that miRNA34a, an inhibitor of Sirt1 expression, is increased in human CKD (27) and, therefore we believe that miRNA34a could downregulate Sirt1 in human CKD.

## SIRT1 and Aging

Experimental evidences show that SIRT1 plays critical roles in age-related pathological changes such as age-related kidney damage (28). Previous studies showed that SIRT1 activity is decreased in the kidneys of aged rodents (29). SIRT1 can protect cells from apoptosis and senescence induced by oxidative stress during aging process. SIRT1 regulates activation of multiple FOXO proteins such as FOXO1, FOXO3, and FOXO4 by inducing their deacetylation in the context of oxidative stress (30). It has been shown that PI3K-Akt, which is upstream signaling pathway for FOXO proteins, is downregulated in aging kidney (31). The aging process is highly associated with systemic hypoxia, which results in apoptosis, metabolism disorder, and abnormal cell cycle. Hypoxia decreases SIRT1 expression to promote FOXO3 acetylation and therefore it inhibits the expression of FOXO3 target genes such as p27Kip1 and Bnip3. In addition, hypoxia causes apoptosis and inhibits the autophagy of senescent cells, leading to the accumulation of senescent cells. In the kidney, SIRT1 also protects the apoptosis of kidney cells by inhibiting Smad7 acetylation, which is mediated by p300 (32). Together, these findings suggest a critical role of SIRT1 in age-related disease of CKD.

## Role of SIRT1 in Vascular Calcification in Chronic Kidney Disease

**Figure 2:**
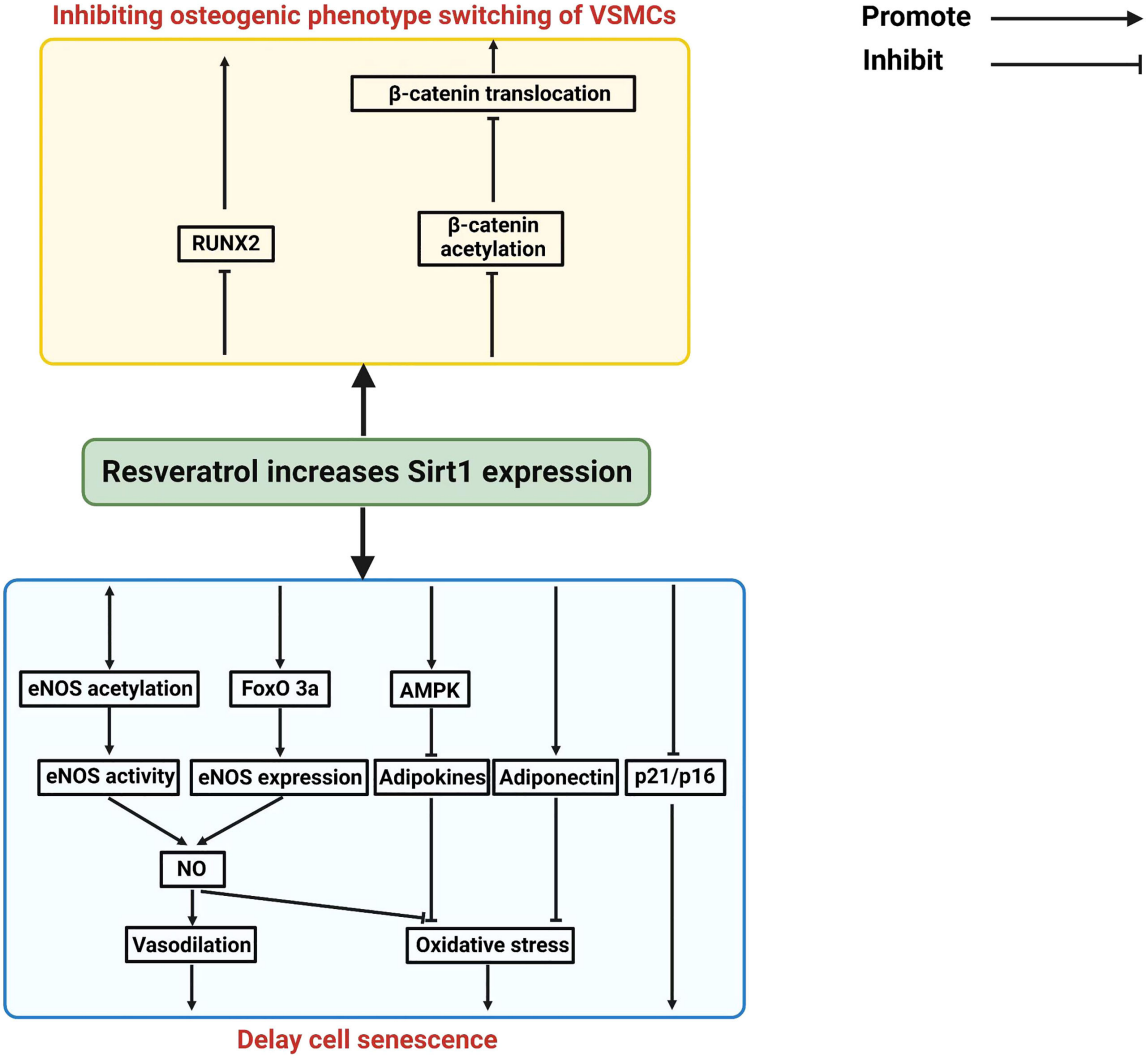
Summary on the mechanisms of Sirt1 in mediating vascular calcifications in CKD patients.

Vascular calcification (VC) is an important pathological finding in patients with CKD, which mediates cardiovascular complication and accompanied by displaying the features of vascular aging (33–36). Vascular calcification is associated with vascular injury such as atherosclerosis, vascular stiffness and vascular aging (37). Vascular calcification can cause the abnormal calcium phosphate crystal deposition in the vessel wall. Increasing osteogenic transition will positively regulate vascular calcification in senescent vascular smooth muscle cells (5). SIRT1 can inhibit vascular calcification (38, 39). A decreased expression of SIRT1 was observed during the development of vascular calcification, and activation of SIRT1 can reduce vascular calcification, suggesting a protective role of SIRT1 in vascular calcification (38, 40, 41). Resveratrol, a pan-activator of SIRT1, ameliorates vascular calcification in animal models (42, 43). Resveratrol activates all Sirt1 isoforms and affects many downstream pathways such as COX and PGC1a (44, 45) and therefore these data do not suggest that this is Sirt1-specific. More specific Sirt1 agonists have been described and future studies are required to confirm whether more specific Sirt1 agonists could also improve vascular calcifications (46–48). Spermidine (Spd) can upregulate SIRT1 and inhibit ER stress, and thereby alleviating vascular calcification in CKD. When the expression of SIRT1 is decreased, the inhibitory effect of Spd on vascular smooth muscle cell calcification is also abolished, suggesting that SIRT mediates the protective effect of Spd on the progression of vascular calcification. In addition, a large body of evidence suggest that SIRT1 plays a critical role in inhibiting the senescence of vascular smooth muscle cells and endothelial cells (49, 50). Also, SIRT1 regulates RUNX2 deacetylation to affect its transcriptional regulation in hyperglycemic conditions (51). In endothelial cells, SIRT1 and eNOS/NO can crosstalk each other to increase their antioxidant and anti-inflammatory property. SIRT1 also inhibits p16 and p21 expression to reduce vascular smooth muscle cells (VSMCs) senescence. In addition, SIRT1 also inhibits osteogenic phenotypic transdifferentiation of VSMCs through deacetylation of RUNX2 and β-catenin (40). SIRT1 also can prevent adipocytokine release through the activation of AMPK pathway or normalization of adiponectin secretion in perivascular adipose tissues (PVAT). Because of these protective mechanisms of Sirt1 in endothelial cells, VSMCs, and PVAT, SIRT1 activators could be considered as potential drugs to inhibit vascular calcification in CKD patients (9, 52). The **Figure 2** summarizes the potential mechanism of Sirt1 in protecting vascular calcification in CKD patients.

## SIRT1 is a Potential Therapeutic Target of CKD

SIRT1 is known to inhibit renal cell apoptosis, inflammation, and fibrosis and therefore could be considered as a potential therapeutic target. A number of studies suggest that SIRT1 has a cytoprotective effect by reducing cell apoptosis, senescence, and by inhibiting inflammation (32, 53). SIRT1 has anti-fibrosis effect in CKD by interacting with TGF-β1 signaling (32). SIRT1 overexpression abolishes TGF-β1-induced kidney cell apoptosis and renal fibrosis, *via* suppression of CTGF expression (54). NF-*κ*B can regulate senescence and cell cycle-specific gene expression (55). SIRT1 can interact directly with NF-kB p65, resulting in p65 deacetylation and NF-*κ*B inactivation (56). Interestingly, the study indicates that SIRT1 and NF-kB regulates each other in a positive feedback loop (57). These studies suggest that SIRT1 activators should have multiple beneficial effects in kidney cells including anti-apoptosis, anti-inflammation, and anti-senescence. Therefore, Sirt1 activators could be developed as a new drug to treat patients with CKD. In addition, SIRT1 attenuates development of hyperphosphatemia-related vascular calcification *via* inhibiting cell senescence and osteogenic phenotype switching of VSMCs and therefore activation of SIRT1 or restauration of Sirt1 expression could be a reasonable approach to reduce hyperphosphatemia-induced medial calcification in CKD patients (38).

However, most of the studies are performed in Sirt1 deficient animals and these studies can only show the association of Sirt1 deficiency with kidney disease. In order to show whether increased Sirt1 expression after onset of CKD has any beneficial effects, we generated an inducible Sirt1 overexpression mouse mode. In these mice, we show that induction of Sirt1 expression after disease onset still has renal protective effects in mice with DKD and HIV kidney disease (25, 48). In addition, we show that BF175, a novel Sirt1 specific agonist, also reduces kidney injury in these animal models. Our studies suggest that targeting Sirt1 could be a potential new therapy for CKD (25, 48).

## Clinical Perspectives

In summary, SIRT1 induces deacetylation of several key transcriptional factors in regulation of cell apoptosis, senescence, inflammation and fibrosis in the context of CKD. SIRT1 activators could be developed to inhibit the apoptosis and senescence of kidney cells, reduce renal inflammation and oxidative stress, improve mitochondrial function, and reduce renal fibrosis. Therefore, SIRT1 activators could be considered as a new therapy to prevent the development and progression of CKD. In addition, SIRT1 has protective effects against vascular calcification which is a major complication of CKD. Therefore, SIRT1 activators should have beneficial effects for both CKD and its complication.

## Author Contributions

All authors listed have made a substantial, direct, and intellectual contribution to the work, and approved it for publication.

## Funding

YZ is supported by National Natural Science Foundation of China (2019-81973772).

## Conflict of Interest

The authors declare that the research was conducted in the absence of any commercial or financial relationships that could be construed as a potential conflict of interest.

## Publisher’s Note

All claims expressed in this article are solely those of the authors and do not necessarily represent those of their affiliated organizations, or those of the publisher, the editors and the reviewers. Any product that may be evaluated in this article, or claim that may be made by its manufacturer, is not guaranteed or endorsed by the publisher.
